# Small and Large Intestine (II): Inflammatory Bowel Disease, Short Bowel Syndrome, and Malignant Tumors of the Digestive Tract

**DOI:** 10.3390/nu13072325

**Published:** 2021-07-06

**Authors:** Yolanda Ber, Santiago García-Lopez, Carla J. Gargallo-Puyuelo, Fernando Gomollón

**Affiliations:** 1 Instituto Aragonés de Ciencias de la Salud (IACS), Gastroenterology and Hepatology Department, Hospital San Jorge, 22004 Huesca, Spain; ybernieto@gmail.com; 2 Instituto de Investigación Sanitaria Aragón (IIS Aragón), Gastroenterology Department, Hospital Universitario Miguel Servet, 50009 Zaragoza, Spain; sgarcia.lopez@gmail.com; 3 Gastroenterology Department, Hospital Clínico Universitario Lozano Blesa, 50009 Zaragoza, Spain; carlajerusalen@hotmail.com; 4 School of Medicine, University of Zaragoza, 50009 Zaragoza, Spain; 5 Centro de Investigación Biomédica en Red de Enfermedades Hepáticas y Digestivas (CIBERehd), 28029 Madrid, Spain

**Keywords:** inflammatory bowel disease, short bowel syndrome, gastrointestinal tumors, undernutrition, surgery, macronutrients, micronutrients, enteral nutrition, parenteral nutrition, nutritional status

## Abstract

The small intestine is key in the digestion and absorption of macro and micronutrients. The large intestine is essential for the absorption of water, to allow adequate defecation, and to harbor intestinal microbiota, for which their nutritional role is as important as it is unknown. This article will describe the causes and consequences of malnutrition in patients with inflammatory bowel diseases, the importance of screening and replacement of micronutrient deficits, and the main indications for enteral and parenteral nutrition in these patients. We will also discuss the causes of short bowel syndrome, a complex entity due to anatomical or functional loss of part of the small bowel, which can cause insufficient absorption of liquid, electrolytes, and nutrients and lead to complex management. Finally, we will review the causes, consequences, and management of malnutrition in patients with malignant and benign digestive tumors, including neuroendocrine tumors (present not only in the intestine but also in the pancreas).

## 1. Introduction

As part of a topic series, our goal in this narrative review is to summarize key evidence for nutritional issues in a heterogeneous group of diseases affecting the small and large bowel. Although very different in etiology, pathogenesis, and symptoms, these diseases share mild to severe consequences in nutrition, which means that diagnostic and therapeutic approaches should always include a clear nutritional perspective.

## 2. Inflammatory Bowel Disease

### 2.1. Introduction

Inflammatory bowel disease (IBD) mainly includes two entities: ulcerative colitis (UC) and Crohn’s disease (CD). Although they are well-differentiated entities, both share chronic and uncontrolled intestinal inflammation in genetically predisposed patients, triggered by environmental factors that lead to a defective response of the immune system. Malnutrition is common in IBD patients and ranges from 12% to 85%; this wide range is a result of the heterogeneity of series regarding disease type, extension, inflammatory activity, and diverse evaluation methods [1,2,3]. Even though malnutrition is generally more prevalent in hospitalized patients with severe activity, a recent multicenter study showed that the prevalence in patients with IBD who attended outpatient visits was 16%, despite most patients in apparent clinical remission [2]. Undernutrition has a negative impact on the clinical course, the rate of postoperative complications, and mortality [4,5,6].

Signs and symptoms of malnutrition in these patients can range from asymptomatic to weight loss, growth failure in children, bone disease, or clinical manifestations of micronutrients deficiency (see Table 1) [7]. Malnutrition is a possibility in all clinical encounters with IBD patients, and nutrition evaluation tools should be part of standard clinical evaluation.

### 2.2. Causes of Malnutrition in IBD (Pathogenesis)

Multiple factors can contribute to the appearance of malnutrition in patients with IBD (summary Table 2).

#### 2.2.1. Poor Dietary Intake

The decrease in food intake is one of the factors that decisively contributes to malnutrition in IBD patients and may be due to several causes. Anorexia linked to inflammation appears to be a major contributor; high levels of inflammatory mediators such as IL-6 and TNF-α are cachectogenic [8,9]. Other causes are dietary restrictions imposed by the patient, the patient’s environment, or even by medical advice; presence of nausea or vomiting, abdominal pain, or obstructive symptoms; and side effects of medications. Several studies show that most IBD patients modify their dietary habits after diagnosis [2,10,11].

#### 2.2.2. Increase Nutrient Malabsorption

Malabsorption is common in CD patients with involvement of the small intestine, especially in those suffering extensive resection surgeries of the small intestine. Mucosal inflammation rarely produces clinically significant nutrient malabsorption, except in cases with great involvement of the jejunum. However, bile acid malabsorption is frequent in CD patients. The ileum is involved in the great majority of cases and can produce alterations in lipid digestion and steatorrhea, changes in intestinal motility and the microbiota, and it is one of the factors responsible for diarrhea [12]. Likewise, steatorrhea may be the consequence of altered secretion of pancreatic enzymes, described in up to 80% of CD patients in some series [13].

#### 2.2.3. Increased Protein Loss

Protein loss occurs through capillary leakage from the inflamed intestinal mucosa. This can contribute to hypoalbuminemia, although systemic inflammation is the main driver. The levels of inflammatory cytokines, including TNF-α, IFN-γ, IL-1β, IL-6, and IL-1, are elevated in the intestines of IBD patients. This cytokine pattern can disturb intestinal barrier function, in particular, the excess of TNF-α [14]. The more severe the inflammation, the more intense the protein loss in the intestinal lumen, and this can also affect drugs if they are proteins, as in the case of monoclonal antibodies.

#### 2.2.4. Increased Metabolism

Increased basal metabolism due to increased pro-inflammatory cytokines contributes to malnutrition in these patients. Increased lipid oxidation and increased thermogenesis induced by the diet have also been described. This is increased in patients with both active and remission CD, which helps to explain the low weight and the decrease in fat mass frequently present in some patients [15,16].

### 2.3. Impact of IBD on Nutritional Status: Macro and Micronutrients

#### 2.3.1. Macronutrients

A marked deficiency of macronutrients is uncommon. Fat malabsorption can appear in up to 30% of CD patients and can cause weight loss and steatorrhea [17]. It is due to multiple factors, such as inflammation of the small intestine, bile acid malabsorption secondary to ileal resection, the activity of IBD, and bacterial overgrowth [18,19].

#### 2.3.2. Micronutrients (Vitamins and Minerals)

Micronutrient deficiencies are more frequent in CD patients than in UC. The risk of certain deficits depends on the location and activity of the disease since some micronutrients are absorbed in specific segments of the gastrointestinal tract, but symptoms rarely develop (except for iron, folate, vitamin B12, and zinc) [3]. Table 3 summarizes micronutrients deficiencies and supplementation recommendations.

The most prevalent micronutrients deficiencies are outlined below.

(a) Vitamin D

Vitamin D deficiency is common. For instance, a recent report found a prevalence of 55% in UC and 58% in CD [20]. Risk factors for the development of vitamin D deficiency are a history of intestinal resection, stenosing phenotype in patients with CD, extensive colitis in patients with UC, early corticosteroid treatment, non-Caucasian race, inadequate exposure to sunlight, and decreased intake of dairy products [21,22]. Its deficit has been associated with a more aggressive course of IBD and an increased risk of surgery and hospitalizations, although randomized, prospective studies are sparse [22].

(b) Vitamin B12

The prevalence of vitamin B12 deficiency in CD ranges from 5% to 38% [20,23]. It is the consequence of various mechanisms such as resection of the terminal ileum, inflammatory damage to the ileum, bacterial overgrowth, pancreatic disease, and, rarely, autoimmune gastritis. Cobalamin deficiency is associated with a spectrum of clinical manifestations, commonly megaloblastic anemia or neurologic symptoms, which can be severe and irreversible [23]. Evaluation of cobalamin levels is a key part of the monitoring of IBD, especially of any CD patient with previous ileal resection [24].

(c) Iron

Anemia is the most common systemic complication and extraintestinal manifestation of inflammatory bowel disease [25]. It is present in up to 70% of inpatients and 20% of outpatients with IBD [26]. It is the result of the combination of chronic iron deficiency, anemia of chronic disease, and other micronutrients deficiencies (folate, vitamin B12). Iron deficiency may be due to continuous blood loss through the inflamed and ulcerated mucosa, inadequate iron intake, or malabsorption, and often a combination of these [25]. A key mechanism is the impaired absorption secondary to the effect of proinflammatory cytokines in upregulating the production of liver hepcidin (a mediator of iron homeostasis), which blocks ferroportin-1 and leads to intracellular iron sequestration and decreased absorption [20]. The therapeutic approach is complex: controlling inflammation, iron, folate, cobalamin supplementation, and stimulating erythropoiesis, which have different roles in particular cases.

### 2.4. Consequences of IBD on Growth, Bone Mass, and Sarcopenia

#### 2.4.1. Growth

Growth retardation is one of the biggest complications in children with IBD [27]. At the time of diagnosis, 90% of children have weight loss [28]. It appears in both CD and UC, although it is less frequent in the latter. Approximately 20 to 30% of children suffering from IBD will become adults with abnormally short stature, regardless of steroid use [3]. Although the etiology of growth retardation is not totally clear, its origin is multifactorial and is related to malnutrition, disease activity, genetic aspects, and the use of corticosteroids [27]. Growth normality should be a key therapeutic goal in children with IBD, and effective treatment not to be delayed, as irreversible consequences can follow.

#### 2.4.2. Bone Mass

Osteopenia and osteoporosis are common in patients with IBD, affecting almost 50% of patients. In one study, 56% of CD patients and 48% of UC patients had osteopenia, while 15% of CD patients and 18% of UC patients had osteoporosis [29]. Risk factors for decreased bone mineral density include corticosteroid treatment, IBD activity, and the age of the patient, and there is an increased risk in patients diagnosed before the age of 30 [30,31]. Other possible risk factors are vitamin D and K deficiencies, a low BMI, genetic factors, CD of ileal or ileocolic location, and history of proctocolectomy [32,33].

#### 2.4.3. Sarcopenia

The fat-free mass (lean mass) of IBD patients is lower than that observed in healthy controls, such that up to 28% of CD patients and 13% of UC patients have a CD fat-free mass diminished [34]. The decrease in lean mass is the consequence of increased protein catabolism secondary to IBD activity, decreased physical activity, treatment with systemic corticosteroids, and, to a lesser extent, inadequate protein intake [35]. Loss of 10% of lean mass is associated with poorer wound healing and a higher rate of infection after surgery [36,37]. This loss leads to the presence of fatigue and a worse quality of life.

### 2.5. Assessment of Nutritional Status in IBD

Identifying patients with malnutrition or at risk of developing it is important, due to the implications that this entails. Patients with IBD frequently present alterations in body composition, in the distribution of fat-free mass and fat mass, despite presenting a body mass index (BMI) within normality [38]. For this reason, the definition of malnutrition in these patients should not be restricted to a low BMI, but rather a comprehensive assessment of nutritional status should be carried out, including clinical, anthropometric, and body composition dimensions. Nutritional evaluation in IBD patients should include a complete medical history and exhaustive physical examination, general laboratory tests including micronutrients, and the use of tools to detect malnutrition [7].

#### 2.5.1. Medical History

It is important to find out if there is loss of appetite and/or weight as well as taste disturbances; therefore, doctors should calculate IBD activity indices and specifically ask about symptoms of abdominal pain, nausea, and vomiting. History should also include a record of recent admissions, previous surgeries, chronic illnesses, and infections as well as a registry of medication and alternative therapies, food allergies, and intolerances. In this section, we could include the dietary evaluation, whose main objective is to identify appropriate and actionable areas of change in the diet and lifestyle of the patient [7].

#### 2.5.2. Physical Examination

A general physical examination should be performed, adding weight, height, and calculation of the BMI. Doctors should reflect on if there is a loss of subcutaneous fat or muscle mass. Anthropometric indices, such as the triceps and subscapular fold measurements, are indicators of body and muscle fat [39].

#### 2.5.3. Analytics

A complete blood count and general biochemistry are recommended, where albumin, prealbumin, and transferrin are requested, although in patients with IBD these values may be altered by the inflammatory activity of the disease without the patient presenting a true state of malnutrition. In addition, to detect micronutrient deficiencies, it is advisable to measure the metabolism of iron, vitamin D, vitamin B12, folic acid, and, depending on the case, other micronutrients such as zinc, magnesium, copper, or selenium [7].

#### 2.5.4. Tools for the Detection of Malnutrition

There are several tools to analyze body composition (total body water, fat mass and fat-free mass, bone mass, skeletal muscle, visceral organs and brain), such as the measurement of total body water by isotopic dilution, bioimpedance, dual absorption of X-ray, and measurement of total body cell mass based on the calculation of total body potassium, computed tomography, and magnetic resonance imaging.

Several tools to screen and assess nutrition exist, but further validation of these tools in patients with IBD is required before any specific tool can be recommended for routine clinical practice [20,40]. Due to its simplicity, accessibility, and speed of application, we recommend the subjective global assessment (SGA). Obtained from the anamnesis and physical examination, with this tool it is possible to classify the nutritional status of the patient as well-nourished, moderately malnourished, or severely malnourished. This tool has shown greater sensitivity and specificity for predicting complications associated with malnutrition than other nutritional risk assessment scales [41,42].

### 2.6. Indications for Parenteral and Enteral Nutrition in IBD

Adequate nutritional support, in addition to improving nutritional status, can positively influence the immune response and exert an anti-inflammatory effect on the intestine [43]. Therefore, diet can play an important role in the primary treatment of IBD and as a supportive measure to prevent or treat nutritional disorders. The concept of intestinal rest in a patient with active IBD should be eliminated from routine clinical practice [44].

As a general rule, enteral nutrition (EN) is preferred to parenteral nutrition (PN), as long as there is no contradiction in its use since it is more physiological, cost-effective, and presents fewer complications [45]. Table 4 summarizes indications for PN or EN in IBD patients.

#### 2.6.1. Enteral Nutrition

The primary goals of EN therapy are the prevention and treatment of undernutrition, improvement of growth and development in children and adolescents, and improvements in quality of life [46].

Commercially available nutritional supplements include elemental (free amino acids), semi-elemental (oligopeptide), or polymeric (whole proteins) diets presented as easily assimilated liquid formulas and differing essentially in their protein content. The selection of one type or another is based on individual preferences, patient tolerance, as well as availability and cost.

Crohn’s disease: In children with active CD, enteral nutrition is considered first-line therapy. In contrast, in adults with active CD, enteral nutrition as the sole and first therapy is rarely used; in fact, it is used only when there are formal contraindication for drugs, previous failure of multiple treatments for the acute phase, or for patients who decline other drug therapy [47]. Enteral nutrition and drugs are used in malnourished patients as well as in patients with inflammatory bowel stenosis. In clinical remission and in the absence of nutritional deficits, the benefit of enteral nutrition or supplements has not been demonstrated.

Ulcerative colitis: In active UC (children or adults), enteral nutrition is not recommended as a primary treatment for active ulcerative colitis, but it is recommended as a supplement if the patient has malnutrition or inadequate nutritional intake, and it can be of special help in the management of severe, hospitalized cases [48].

#### 2.6.2. Parenteral Nutrition

PN is indicated for patients who are malnourished or at risk of becoming malnourished and who have an inadequate or unsafe oral intake, a non-functioning or perforated gut, or in whom the gut is inaccessible. Specific reasons in patients with CD include an obstructed gut, a short bowel, often with high intestinal output, or an enterocutaneous fistula [49].

### 2.7. Nutritional Support in Specific Settings Related to IBD

#### 2.7.1. Children and Adolescents

Multiple studies in pediatric CD patients conclude that exclusive enteral nutrition (EEN) is as effective as corticosteroid treatment in inducing remission of the disease [50,51,52,53]. In addition, unlike corticosteroids, EEN promotes mucosal healing and improves bone growth and density. Its mechanism of action is multifactorial: exclusion of antigenic components from the oral diet, trophic and anti-inflammatory effects, restoration of the epithelial barrier, and favorable changes in the intestinal microbiota [48].

#### 2.7.2. Stenosis

The CD patient with stenosis frequently needs nutritional support to avoid malnutrition [54]. The following guideline is recommended:

1. Patients with asymptomatic radiological stenosis: restrict the intake of insoluble fiber in food.
2. Symptomatic strictures: soft or semi-liquid diet, with enteral supplements as needed for avoiding malnutrition.
3. Occlusive symptoms: EEN using a probe distal to the occluded segment (in proximal stenoses). In the presence of complete occlusion, PN is indicated.

#### 2.7.3. Fistula

Patients with CD can develop fistulas, and the correction is usually surgical, but some selected cases may respond to treatment with immunomodulators or biologics. In these cases, they can benefit from EN [55]. Low-output distal fistulas (ileum or colon) may respond to this treatment [56,57]. High-output or proximal fistulas are subsidiary to PN [58,59].

#### 2.7.4. Severe Ulcerative Colitis

Fulminant toxic ulcerative pancolitis presents with marked anorexia and hypercatabolic state and requires artificial nutrition, in these cases generally PN. However, in patients with a severe flare but without toxicity, they can be fed orally since fasting has not been shown to present any advantage over an oral diet [55].

#### 2.7.5. Stoma with Severe Diarrhea

Water depletion and electrolyte loss are common in the first 3–4 weeks of a jejunostomy or high-output ileostomy. In these patients, they require hypotonic fluid restriction, sodium-enriched diets, or EEN [60,61].

### 2.8. Nutritional Support in Perioperative Management

Despite the emergence of new treatments in IBD, a large proportion of patients will undergo surgery. Although this has been reduced in recent years [62], at least half of patients with Crohn’s disease and a quarter of patients with ulcerative colitis will require surgery at some point in their lives [62,63]. Perioperative malnutrition is associated with a higher rate of complications, increased hospital stay, costs, and mortality [4,6].

We should remember that the surgery itself, like any other injury or damage, causes a series of reactions that include the release of stress hormones and inflammatory mediators such as cytokines. These cytokines trigger the systemic inflammatory response syndrome that causes a catabolic state, increasing the use of protein and energy, and in turn producing weight loss and reduction in muscle mass. Macronutrients are mobilized from fat tissue and musculoskeletal tissue reserves to redistribute to other organs, causing malnutrition in a few days [64]. In summary, surgery alone can be a cause of malnutrition or aggravate a previous situation.

The risk of developing postoperative malnutrition varies individually and depends on several factors: the existence of malnutrition before the intervention, the reason and complexity of the surgery performed, the degree of postoperative hypermetabolism, and the patient’s ability to cover energy requirements [60].

#### 2.8.1. Consequences of Malnutrition in Surgical Patients

Malnutrition leads to a dysfunction of the immune system by altering the activation and production of complement, bacterial opsonization, and the function of neutrophils, macrophages, and lymphocytes [65]. Therefore, one of the most direct consequences of malnutrition in patients undergoing surgery is the increase in infections. The relationship between infection and malnutrition is reciprocal. As it has been commented above, malnutrition leads to alterations in humoral and cellular immunity, causing an increased risk of infections; in turn, an infection produces a catabolic state, promoting malnutrition [66]. In addition, malnutrition produces other frequent direct consequences such as poor wound healing and an increase in pressure ulcers. Malnutrition prolongs the inflammatory phase (the first phase of healing in which thromboxane is immediately released, A2 and prostaglandin 2α, from the cell membrane causing vasoconstriction and cessation of bleeding), followed by a decrease in fibroblast proliferation and alterations in collagen synthesis [67].

#### 2.8.2. Key Aspects of Perioperative Care

Following the recommendations of the European Society for Clinical Nutrition and Metabolism (ESPEN) of perioperative care, from a metabolic and nutritional point of view, the key aspects are outlined below [60].

1. Integration of nutrition into the overall management of the patient: Malnutrition has a negative impact on the clinical course, increased complications, and mortality, and its correction has been associated with the reduction in complications in patients with IBD. In a meta-analysis that included five studies with a total of 1111 patients with Crohn’s disease, the patients who received preoperative nutritional support had a lower rate of complications compared to the group that did not receive them (20% vs 61.3% OR 0.26 *p* < 0.001) [68].
2. Avoidance of long periods of preoperative fasting: Fasting from the night before the intervention is unnecessary. If the patient does not present a risk of aspiration, they can drink liquids up to 2 h before anesthesia, and 6 h before in the case of solids [69]. Since the implementation of these new recommendations, there has not been an increase in the incidence of aspiration, regurgitation, or morbidity and mortality.
3. Re-establishment of oral feeding as early as possible after surgery: In most patients, an oral diet can be started in the first hours after the intervention. Restarting oral nutrition early is associated with a significant decrease in complications, mortality, and hospital stay [70].
4. Metabolic control: Insulin resistance develops within minutes of surgery, causing protein loss and alterations in glucose metabolism with rapid release of glycogen in the liver, and similarly occurs in muscle, where the reserves of energy are also depleted. Complications associated with this insulin resistance include infections and cardiovascular problems. Its prevention and treatment is an effective measure to reduce complications after major surgery. ERAS (Enhanced Recovery After Surgery) programs and goals, such as preoperative glucose administration, minimally invasive surgery, and pain control, reduce insulin resistance during surgery by up to 50% [71].
5. Reduction in factors that exacerbate stress-related catabolism or impair gastrointestinal function: It has been shown that measures to reduce the stress of surgery can minimize catabolism and support anabolism, throughout the surgical process, allowing better and faster recovery in patients, even after major surgical operations [72]. ERAS protocols can be used, which combine a series of measures to minimize stress and facilitate the return of function: these include preoperative preparation and medication, fluid balance, postoperative anesthesia and analgesia, pre- and postoperative nutrition, and mobilization [60].
6. Early mobilization to facilitate protein synthesis and muscle function: Physical exercise is the fundamental pillar both in the prevention and in the treatment of sarcopenia. Resistance training improves body composition, leading to an increase in lean body mass and a decrease in fat, including visceral fat, a major risk factor for the development of metabolic syndrome, diabetes, and chronic inflammation [73].

#### 2.8.3. Nutritional Management

Once the nutritional status of the patient has been assessed, nutritional support (preferably enterally) would be indicated when one or more of these conditions are met [60]:

1. Malnutrition has been detected before the operation.
2. It is expected that the patient will not be able to resume an oral diet for the next 7 days.
3. It is estimated that the oral diet will not be able to cover 60–70% of the requirements in the following 10 days.

ESPEN’s nutritional strategies are as follows [60,74]:

1. Patients with IBD are at nutritional risk and therefore should be screened for malnutrition.
2. In general, the energy requirements of patients with IBD are similar to those of the healthy population.
3. Protein requirements increase in active IBD, and intake should be increased (1.2–1.5 g/kg/d in adults) with the recommended intake in the general population. In remission, the protein intake should not be increased.
4. Patients with IBD should be regularly evaluated for micronutrient deficiencies, and specific deficits should be corrected.
5. If the energy protein requirements cannot be covered with an oral diet, oral nutritional supplements will be considered.
6. If the requirements are still not met despite oral nutritional supplements, exclusive enteral tube nutrition is recommended.
7. Patients with serious nutritional risk may benefit from perioperative nutrition, even if it entails a delay in the surgical act. In these cases, enteral nutrition, either by oral nutritional supplements or exclusive enteral nutrition, is preferable to parenteral nutrition, unless there is severe dysfunction of the digestive tract.
8. In the case of a surgical emergency, nutritional support will be carried out postoperatively.
9. Mixed nutrition (enteral and parenteral nutrition) will be performed when at least 60% of the requirements cannot be covered by the enteral route.
10. Exclusive parenteral nutrition is only indicated when enteral nutrition is not feasible or contraindicated, as in cases of intestinal obstruction or ileus, severe shock, intestinal ischemia, high-output fistula, or severe intestinal bleeding.
11. For Crohn’s disease patients who have been deprived of nutrition for many days, standard measures and precautions should be taken to prevent refeeding syndrome, especially regarding phosphate and thiamine.
12. In patients with Crohn’s disease and prolonged intestinal failure, parenteral nutrition is mandatory, at least in the early stages of intestinal failure.
13. In most patients treated for IBD, normal oral food intake or enteral nutrition can be started shortly after surgery.
14. In patients with Crohn’s disease of more than 20 cm from the terminal ileum, with or without preservation of the ileocecal valve, vitamin B12 replacement should be carried out.

## 3. Short Bowel Syndrome (and Intestinal Failure)

### 3.1. Introduction

Short bowel syndrome (SBS) is defined as malabsorption due to the loss of enterocytes secondary to congenital absence, resection of large portions of the small intestine, or another disease that could cause an intestinal function defect. This loss leads to the inability to absorb sufficient liquid, macronutrients, and micronutrients. Intestinal failure is a subgroup of SBS with a decrease in the intestinal function below the minimum necessary for the absorption of macronutrients and/or water and electrolytes, and it requires intravenous support to maintain health and/or growth [75]. The main causes of SBS are shown in Table 5.

The spectrum of the clinical disease is widely variable from only single micronutrient malabsorption (see Table 1) to complete intestinal failure (oral intake intolerance, diarrhea, and severe micro and macronutrient deficiency).

Patients with SBS experience reduced long-term survival compared with the general population. The youngest patients are initiated on home parenteral nutrition (HPN), and the primary disease is a much more common cause of mortality, where the risk is greatest during the first 2 years of HPN [76].

### 3.2. Pathophysiology of Short Bowel Syndrome

The severity and type of malabsorption is determined by several factors: the loss of absorptive surface area (the most important); the rapid intestinal transit that decreases nutrient-enterocyte contact time, and therefore the absorption decreases too; colon removal; and small intestinal dysbiosis secondary to alteration in motility and loss of the ileocecal valve [77,78].

Although the length to produce a SBS is not exact, the nutrient absorption is preserved until the length of the functional intestine is less than 200 cm. The small intestine enterocytes lining is uniform, but a proximal-to-distal gradient exists in morphology and function; most macronutrients are absorbed in the proximal 100–150 cm of the intestine [79] The loss of different anatomical sites may result in specific nutrients deficiency (see Table 6). In addition, the loss of the distal ileum and ileocecal valve causes the lack of inhibitory hormones signals that result in intestinal rapid transit, gastric hypersecretion, and dumping syndrome [80].

Citrulline, a nonessential amino acid produced by enterocytes, may function as a marker of intestinal failure. In patients with SBS syndrome, citrulline levels were lower than in controls, and their values correlated with PN dependence at 2 years postresection (plasma levels of <20 µmol/L) [81,82].

Classification depending on the anatomic resection:

1. End-jejunostomy: the ileum and colon completely removed. These patients present with accelerated gastric emptying and bowel transit due to reduced hormonal secretion of polypeptide YY and enteroglucagon 1 and 2. In addition, the increase in excretion of water and electrolytes leads to a high risk of dehydration immediately after surgery and induces hypomagnesemia, hypotension, and renal failure [83].
2. Jejunocolonic anastomosis: jejunoileal resection with the preservation of the colon. These patients can reabsorb unabsorbed fluids in their colon. On the other hand, most of the ileum has been removed; therefore, the clinical course is dominated by malnutrition, diarrhea/steatorrhea, and vitamin deficiencies [83].
3. Jejunoileal anastomosis: jejunal resection leaving ≥10 cm of the terminal ileum and the colon intact. These patients usually do not need nutritional support [83].

We can distinguish three phases according to the time in the development of the SBS:

1. Acute phase: This phase starts immediately after resection and lasts for 3 to 4 weeks. It is characterized by huge enteric losses due to an increased gastric secretion as a consequence to the loss of hormonal feedback signals. It is important to avoid dehydration, acute kidney failure, acid/base imbalance, and electrolyte abnormalities [84].
2. Adaptation phase: The intestines undergo an innate process of functional recovery, and this phase lasts 1–2 years. The changes increase the absorptive surface, while the time of intestinal transit decreases to optimize the time available for absorption. In brief, there are structural and functional adaptations, but it is necessary to promote them by the presence of nutrients within the gut lumen [83].
3. Failure phase: This phase is characterized by an inability to withstand oral or enteral feeding and requires parenteral nutrition. It can be reversible (intravenous nutritional support over months or years) or irreversible (lifelong PN).

In conclusion, the extent of nutrition deficiency can be influenced not only by the segment of the intestine that is resected but also by existing residual disease, the presence of small bowel-colon continuity, and time since resection.

### 3.3. Consequences on Metabolism, Liver, and Kidney Function

The complications and consequences in SBS patients can occur as a consequence of SBS as well as complications of long-term HPN.

#### 3.3.1. Metabolism Complications

(a) D-lactic acidosis: A rare and severe neurological syndrome, manifested by lethargy, confusion, and unexplained acidosis. It occurs in some SBS patients with an intact colon. SBS leads to an increased load of undigested carbohydrates to the colon as well as *Lactobacillus bacteria* that produce an excessive fermentation of carbohydrates and increase their production and absorption of D- lactic acid [85].
(b) Metabolic bone disease: Low bone mineral density with metabolic bone disease is commonly reported in patients receiving HPN. The pathogenesis is related to several factors that may be HPN-related and/or related to a patient’s underlying disease. In SBS patients with poor nutrient absorption, particularly fat malabsorption, vitamin D deficiency may play a role [86].
(c) Iron deficiency anemia: Iron deficiency anemia has been reported in patients receiving HPN, and this deficiency varies from 32% to 55% [87,88].
(d) Cholelithiasis: It can occur in up to 44% of patients with SBS [89]. The loss of bile salts, due to the absence of ileal reabsorption, combined with a decrease in the emptying of the gallbladder, caused by the reduction in the oral intake of nutrients, and the decrease in the production of cholecystokinin promote the formation of gallstones. Risk factors include the use of PN, resection of the terminal ileum, and an intestinal remnant <120 cm [89].

#### 3.3.2. Liver Complications

Intestinal failure-associated liver disease (IFALD): IFALD is characterized by either liver steatosis or cholestasis and may develop in patients on long-term HPN for chronic intestinal failure. It covers the complete spectrum of conditions from elevated liver enzymes, steatosis/steatohepatitis, cholestasis, and fibrosis to cirrhosis end-stage liver disease [90,91]. The pathogenesis of IFALD is partly understood and most likely is multifactorial: overload lipids, overload glucose, continuous PN infusion, nutrient deficiency (vitamin E, choline, taurine, essential fatty acid), ultrashort bowel, lack of oral/enteral feeding, no colon in continuity with remnant small bowel, lack of ileocecal valve, disruption of enterohepatic circulation, recurrent sepsis, and small intestinal bacterial overgrowth [91].

#### 3.3.3. Kidney Function

Oxalate nephropathy: The saponification of calcium by fats allows free oxalate to be transported to the colon, where it is absorbed and concentrated in the kidney. There it combines with calcium and precipitates within the ureter and renal pelvis [89].

### 3.4. Management

Because of the heterogeneity of the patient population with SBS, therapeutic options should be individualized for each patient. The management is complex (resume in Table 7) and includes nutritional, medical, and surgical therapy.

#### 3.4.1. Nutritional Therapy

The most important aspect of dietary treatment is to stimulate hyperphagia, except in patients with proximal jejunostomy, to which it is advisable to reduce intake, especially of liquids, because they are net secretors, and as intake increases, fecal and fluid losses increase. Immediately after resection, the patient requires an intravenous liquid resuscitation, then PN, and progressively the introduction of oral liquids and food. Initially PN and oral/enteral nutrition may coincide. Several enteral nutritional formulations exist for correcting nutritional management, and doctors should not forget to detect, correct, and monitor the deficit of macro and micronutrients. The rate of PN dependency at 1, 2, and 5 years was reported as 74%, 64%, and 48% [92]. We recommend expanding nutritional management in these patients, with the guidelines of the European Society for Parenteral and Enteral Nutrition (ESPEN), published in 2016, 2017, and 2020, for specific recommendations on the nutritional management in surgery, chronic intestinal failure, home enteral nutrition, and home parenteral nutrition [60,61,93,94]

#### 3.4.2. Drug Therapy

Medical treatment aims to improve diarrhea, which is the most common symptom and most affects the quality of life of these patients, and to reduce the frequency and volume of parenteral nutrition.

To improve diarrhea, loperamide 4–16 mg/d can be used, even doubling that dose, sometimes in combination with diphenoxylate-atropine or codeine. Transdermic clonidine is a new treatment for patients with SBS with intact colon. It is an α 2-adrenergic agonist and decreases the volume and weight of feces [95]. Octreotide is an alternative for select patients in which high-volume diarrhea is resistant to conventional treatment, generally due to dumping syndrome [96]. Gastric hypersecretion that occurs initially after intestinal resection can be controlled by administering a proton pump inhibitor orally or intravenously for the first 6 months after resection, although it has modest effects on fluid loss [80,97]. We can also use antibiotics such as rifaximin or metronidazole if the patient has diarrhea and/or abdominal discomfort secondary to dysbiosis.

To reduce the frequency and volume of PN, the use of teduglutide, a glucagon-like peptide-2 analogue, shows significant effects on reducing intestinal wet weight excretion and improving wet weight absorption in SBS patients. In SBS patients with an end jejunostomy, teduglutide significantly increased villus height, crypt depth, and mitotic index [98], and 63% of patients taking 0.05 mg/kg/d of teduglutide subcutaneously achieved a ≥20% reduction in weekly HPN volume from baseline to week 20 and maintained it to week 24 [99].

#### 3.4.3. Surgical Procedures

Surgical procedures can be subdivided into autologous intestinal reconstruction procedures and small bowel transplantation. Two main autologous intestinal reconstruction procedures applied today are the Longitudinal Intestinal Lengthening and Tailoring (LILT) and the Serial Transverse Enteroplasty (STEP) [100,101]; both aim to lengthen the intestine. The LILT technique divides the dilated bowel, creates two hemiloops, and anastomoses the hemiloops in an end-to-end fashion, thereby doubling the bowel length. The STEP procedure is less complex, in which a linear surgical stapler is applied from alternating and opposite directions along the intestine’s mesenteric border to incompletely staple and divide the dilated intestine. A comparative study between the two techniques concluded that the outcome after STEP seems to be more favorable [102]. These procedures propose to increase the absorptive area, slowing down the intestinal transit and reducing bacterial overgrowth. Intestinal transplantation should be suggested to a very select subset of SBS patients with severe and irreversible complications of long-term PN.

#### 3.4.4. Future Treatments

Recent advances in 3D in vitro culture technologies, such as organoids, in the near future could be another potential way to treat SBS. It could be an alternative, before performing an intestinal transplant, which carries a serious risk of severe allogenic reaction. In a new study published in *Nature*, researchers use organoids to generate a functional small intestinal colon that has potential applications in the treatment of SBS [103,104].

## 4. Malignant Tumors of the Digestive Tract

### 4.1. Introduction

Colorectal neoplasms are very common, while small bowel tumors are infrequent. The main functions of the colon consist in absorbing water, to allow adequate defecation, and to harbor intestinal microbiota, for which the nutritional role is as important as it is unknown. The small intestine is key in the digestion and absorption of nutrients. We will briefly review the nutritional causes and consequences of intestinal neoplasms along with neuroendocrine tumors (present not only in the intestine but also in the pancreas). We also include benign small bowel neoplasms and describe specifically common causes and consequences of cancer-associated malnutrition.

### 4.2. Colon and Rectal Tumors

Colorectal cancer (CRC) is the third leading cause of cancer death in the Western world [105,106]. The vast majority are adenocarcinomas. Their frequency is increasing, and the Western lifestyle seems largely responsible [107]. Although we do not know exactly, some of the factors involved seem to be tobacco, sedentary lifestyle, and of course diet. In this sense, a recent umbrella analysis of 45 meta-analyses found a significant association between a low risk of CRC and the consumption of high amounts of dietary fiber, calcium, and yogurt and the low consumption of alcohol and red meat. The study did not show a clear relationship with other foods such as processed meat or with specific dietary patterns, with variable results between studies [108]. It is estimated that around half the risk of CRC is based on modifiable risk factors, including diet, and consequently dietary recommendations could be established to minimize it [108,109]. Many of these positive effects of the diet, particularly those of fiber, would be exerted through its relationship with the microbiota and its derived products [106].

The main nutritional impact of CRC may be malnutrition associated with neoplastic disease, in addition to the usual iron deficiency anemia due to chronic bleeding. CRC surgery does not usually produce significant nutritional problems, except when performing a very extensive colectomy, which may involve significant diarrhea with electrolyte and water loss and dehydration. Left hemicolectomy usually has few nutritional consequences, and right hemicolectomy occasionally produces diarrhea due to malabsorption of bile salts or bacterial overgrowth associated with resection of the ileocecal valve. The impact of these surgeries, as well as the subsequent recovery, varies greatly between individuals. We actually do not know the impact of CRC or its surgery on the patient’s microbiome, its potential consequences, and its relationship with diet.

### 4.3. Small Bowel Tumors

Small bowel tumors, benign and malignant, are rare (less than 3–5% of all gastrointestinal neoplasms) [110]. The etiology is unknown, but in some cases, factors such as persistent inflammation or diet may be involved. Malignant are more common than benign tumors and include adenocarcinoma, lymphoma, carcinoid tumor, and GIST (gastrointestinal stromal tumors). The most common benign tumors are adenomas, followed by lipomas and leiomyomas. Exceptionally, other benign and malignant tumors are possible, even metastases from other adenocarcinomas or another neoplasm [111]. Symptoms of small intestine neoplasms are not specific and will depend on the benign or malignant nature, extent, location, specific type, and eventual complications. They can be asymptomatic (being diagnosed in laparotomy for another reason, for example) or produce different symptoms, secondary to acute or chronic bleeding or to a complete or partial obstruction of the intestinal lumen. From the point of view of its nutritional consequences, they are also not specific, with the exception of neuroendocrine tumors. The diagnosis of small bowel neoplasms requires a high clinical suspicion grade; it is not simple, and it is usually late, which explains that in some cases extensive surgical resections are necessary, compromising normal intestinal function. Table 8 summarizes the most common types of small bowel tumors and their main characteristics.

Due to their nutritional and metabolic consequences, we specifically mention neuroendocrine tumors: carcinoid tumors, which usually settle in the bowel, and pancreatic neuroendocrine tumors. They originate from the neuroendocrine system cells and present diffusely, especially in the bowel, pancreas, and bronchial tract. Its course is usually more benign than other neoplasms and may or may not produce hormones responsible for specific alterations, in addition to nonspecific malnutrition. Carcinoid tumors develop from the enterochromaffin cells of the intestinal wall and are the most frequent tumors in the terminal ileum, although other locations are possible [112]. Those tumors may produce a specific clinical picture, named carcinoid syndrome, due to the release of histamine or other peptides. It consists of chronic diarrhea, facial flushing, bronchospasm, and carcinoid-heart disease. Chronic uncontrolled diarrhea leads to dehydration, weight loss, and electrolyte and trace mineral disturbances. Sometimes the syndrome is triggered by the consumption of alcohol and some food that contain amines, such as aged cheeses, smoked, salted or pickled meat or fish, yeast extract, brewer’s yeast, broad beans, and soy products. The high consumption of serotonin, synthesized from tryptophan, can lead to a niacin deficiency that can cause pellagra, characterized by skin and mucous lesions and mental confusion. Furthermore, vitamin D and B12 deficiencies due to malabsorption and bacterial overgrowth are very often present, whether or not clinically apparent.

The rest of the neuroendocrine tumors of the digestive tract and pancreas may produce other hormones and corresponding specific symptoms (Table 9) [113,114]. The most relevant are metabolic/nutritional, affecting electrolytes, fat-soluble vitamins, or trace elements, which can be associated with alterations secondary to treatment, especially pancreatic insufficiency after some surgeries.

### 4.4. Causes of Cancer-Associated Malnutrition

Anorexia (loss of appetite) and cachexia (a hypercatabolic state characterized by accelerated loss of skeletal muscle), very common in cancer patients, are the main patient-related causes of malnutrition. There are several mechanisms involved in this malnutrition that depend on the tumor, host response to the tumor, and treatment.

#### 4.4.1. Mechanisms Related to the Tumor

##### Tumor-Related Digestive System Disorders

The existence of digestive cancer can induce mechanical or functional alterations that alter the alimentation of the patient. The clinical manifestations are highly variable, depend on the location of the tumor, and include alterations in food intake (odynophagia, dysphagia), abdominal pain, intestinal obstruction, early satiety, and malabsorption.

##### Tumor-Induced Metabolic Alterations

Mediator molecules of tumor origin could also play a role in cachexia. An example is the “proteolysis inducing factor” (PIF) that could be involved in increased muscle protein degradation, decreased protein synthesis, and weight loss [115,116], or a “lipid-mobilising factor” present in the urine of some patients with cancer, which can stimulate lipolysis, increase metabolic rate, and cause loss of adipose tissue in mice [117].

##### Inflammatory Mediators

Tumor microenvironment (TME) cells, as a complex cellular network, are made up of cancer cells. Various populations of stromal cells (e.g., macrophages, neutrophils, and fibroblasts) have shown a significant role in both cancer progression and cachexia induced by the production of multiple procachectic factors [118]. Various pro-inflammatory cytokines such as tumor necrosis factor -α (TNF-α), IL-6, IL-8, interferon (IFN) γ, and macrophage migratory factor (MIF) have an important role in cancer cachexia [8,9,116,119]. TNF-α can promote most of the abnormalities found during cancer cachexia: loss of weight, loss of appetite, increased thermogenesis, alterations in carbohydrate, protein and lipid metabolism, insulin resistance, and wasting of muscle by the activation of the breakdown of protein [116].

#### 4.4.2. Host Response to the Tumor

##### Psychological Effects

Cancer patients are greatly affected psychologically as a consequence of the cancer diagnosis itself. Some psychosocial factors, such as depression or anxiety, can cause a significant alteration in nutrition [120].

##### Anorexia and Cachexia

Anorexia, defined as loss of appetite, can reach 80% of patients with advanced cancer [121]. It generally results in reduced caloric intake, malnutrition, and weight loss. It can be a consequence of nausea, fatigue, altered taste, depression, pain, and disorders of gastrointestinal motility.

Cachexia is a complex metabolic disorder defined by an accelerated loss of skeletal muscle. The prevalence in palliative care populations varies from 12 to 85% [122], and surely this variation is due to the variety of its definition. They play an important role in its pathogenesis, the activation of cytokines, and for several tumor-derived cachexia-inducing substances, causing changes in body composition and alterations in nutrient metabolism.

#### 4.4.3. Treatment Related

##### Surgery

The surgery itself, like any other injury or damage, causes a series of reactions that include the release of stress hormones and inflammatory mediators such as cytokines, causing malnutrition in a few days [64]. In surgery for digestive tumors, there are more post-surgical alterations that can cause malnutrition: dysphagia, early satiety, dumping syndrome, fistulas, micronutrient malabsorption, diarrhea and electrolytes alterations in the small bowel or colon resections, hyperglycemia or encephalopathy in liver surgeries, and steatorrhea, fat and protein malabsorption, and hyperglycemia in pancreatic surgeries.

##### Chemotherapy

Adverse effects of chemotherapy can negatively affect nutritional status and include anorexia, altered perceptions of taste and smell (food tastes metallic, like cardboard or sandpaper), food aversions (mainly meat), mucositis, constipation or diarrhea, early satiety, abdominal cramping, bloating, and paralytic ileus.

##### Radiotherapy

The gastrointestinal mucosa is highly vulnerable to radiation therapy. Their effect on nutritional status depends on the body area irradiated, quantity and duration, and the individual’s response. It may cause diarrhea, anorexia, nausea, vomiting, abdominal pain, and enteritis and colitis.

### 4.5. Consequences of Cancer-Associated Malnutrition

This type of malnutrition negatively influences the quality of life, prognosis, response to anticancer treatment, and overall survival.

#### 4.5.1. Impact on Quality of Life

Oncology patients suffer from a broad spectrum of clinical conditions resulting from cancer and/or its treatment, which progressively contributes to the decline in their quality on life. Malnutrition is often associated with poor functional status and impaired quality of life due to poorer general health and reduced social functioning [122].

#### 4.5.2. Response to Treatment and Mortality

In patients with digestive cancer, several studies show that moderate-to-severe malnutrition increases the toxicity of chemotherapy as well as decreased overall survival [123,124,125,126].

## 5. Conclusions

This paper tries to explain the nutritional causes and consequences of three digestive diseases that affect the small and/or large intestine. Due to the high prevalence of malnutrition in these patients, even in patients with non-active disease, as in the case of inflammatory bowel disease or as consequence of restrictive surgery, we recommend detecting and treating the deficits they present. Treatment must be individualized, and we have described brief recommendations.

## Figures and Tables

**Table 1:** Symptoms and signs of micronutrient deficiency.

Micronutrients	Signs/Symptoms/Consequences
Iron	Anemia, fatigue, weakness, brittle nails
Vitamin D	Disturbed calcium homeostasis and bone health
Vitamin B12	Anemia, fatigue, neurological effects
Zinc	Impaired healing, disturbed smell and taste, delayed growth in children
Folate	Anemia, fatigue
Calcium	Decreased bone density (risk bone fracture)
Magnesium	Disturbed bone health, muscular cramps, fatigue

**Table 2:** Factors that contribute to malnutrition in IBD patients.

FACTOR	MECHANISM
Poor dietary intake	Inflammation-related anorexia Restrictive diet Medical advice Nausea, vomits and abdominal pain Obstructive symptoms Side effects of medications
Increase nutrient malabsorption	Intestinal inflammation Restrictive surgeries Diarrhea Bacterial overgrowth Bile acid malabsorption
Increase protein loss	Mucosa inflammation Fistulas Bacterial overgrowth
Increased metabolism	Increase pro-inflammatory cytokines Increased diet-induced thermogenesis Corticosteroid treatment Infectious complications

**Table 3:** Micronutrient deficiencies and supplementation recommendations.

DEFICIT	RECOMMENDATION
Vitamin D	Supplement in case of detecting deficit
Vitamin B12	Supplement in case of detecting deficit and in all patients with ileal resection >20 cm
Folate	Supplement in case of detecting deficit
Iron	Iron deficiency anemia To supplement with oral iron if mild anemia, inactive IBD and good oral tolerance To supplement with intravenous iron if; hemoglobin <10 g/dl, active IBD or oral iron intolerance Iron deficiency without anemia: to supplement with oral iron
Zinc	Selective cases: severe diarrhea

**Table 4:** Indications for enteral and parenteral nutrition in IBD patients.

ENTERAL NUTRITION	PARENTERAL NUTRITION
Prevent and treat malnutrition	Malnourished patients or at risk of malnutrition when it is not possible to administer oral or enteral diet
Improve growth and development in pediatric patients	Intestinal perforation
Perioperative nutrition in CD patients with weight loss before surgery and low albumin	Non-functioning intestine
In pediatric CD patients, as first-line treatment to induce remission	CD with intestinal obstruction, short bowel or high-output enterocutaneous fistula

**Table 5:** Causes of SBS.

ADULTS	CHILDREN
Intestinal resection for Crohn’s disease, tumor or trauma Catastrophic vascular accidents (mesenteric ischemia) Chronic intestinal pseudo-obstruction Midgut volvulus Radiation enteritis Refractory sprue Scleroderma and mixed connective tissue disease	Congenital villus atrophy Extensive aganglionosis Gastroschisis Jejunal or ileal atresia Necrotizing enterocolitis

**Table 6:** Specific areas of absorption in the small intestine and colon.

Proximal small intestine	Fat Sugars Peptides and amino acids Iron Folate Calcium Water Electrolytes
Middle small intestine	Sugars Peptides and amino acids Calcium Water Electrolytes
Distal small intestine	Bile acids Vitamin B12 Water Electrolytes
Colon	Water Electrolytes Medium-chain triglycerides Calcium Amino acids

**Table 7:** Management of SBS.

Immediate Postresection	Early Postresection	Late Postresection
Intravenous fluid resuscitation	PN Control of diarrhea Prevention of nutrition deficiencies Progressive introduction of oral liquids and food Monitoring hydration status	Oral intake ± PN Some patients: long-term PN Control of diarrhea Prevention and correction of nutritional deficiencies Continued monitoring hydration status If appropriate: hormonal therapy If feasible: restoration of intestinal continuity If appropriate: surgical procedures to increase length or improve motility Selected patients: intestinal transplantation

SBS: short bowel syndrome, PN: parenteral nutrition.

**Table 8:** Relevant clinical, diagnostic, and nutritional features of small bowel tumors.

**Benign Neoplasms of the Small Intestine**					
	Location	Clinical peculiarities	Diagnosis	Various peculiarities	Nutritional peculiarities
Adenomas	Duodenum, ileum (less common). They are usually unique; when multiple, consider associated polyposis.	Asymptomatic, except if they obstruct the ampulla of Vater and produce obstructive colostasis/jaundice	According to location, endoscopic technics. Evident malignant potential: at diagnosis 50% malignant degeneration (invasive or not).	The most frequent. Potential association with polyposis syndromes, such as familial adenomatous polyposis.	Usually, asymptomatic. Chronic biliary obstruction (unusual) could lead to deficits in the absorption of fat and fat-soluble vitamins.
Lipoma	Mature adipose tissue. No malignant potential.	Asymptomatic in general.	CT and MRI high diagnostic specificity.	In asymptomatic patients with a firm preoperative diagnosis, no treatment.	Not common, not specific.
Leiomyoma	Any location, most common in the jejunum.	Asymptomatic except if they are large: obstruction or less frequently bleeding.	Sometimes they adopt radiological characteristics similar to GIST.	Second in frequency.	Not common, not specific.
**Malignant Neoplasms of the Small Intestine**					
	Location	Clinical peculiarities	Diagnosis	Various peculiarities	Nutritional peculiarities
Adenocarcinoma	Duodenum or proximal jejunum (90% in these locations), originating from an adenoma. Sometimes in the context of genetic syndromes. Consider metastasis from another adenocarcinoma.	Obstruction and/or bleeding (chronic macro or microscopic) and/or obstructive jaundice if they affect Vater’s ampulla.	Endoscopy, imaging tests (these are not specific)	Most common malignant tumor of the small intestine. Possible association with polyposis (familial adenomatous, Lynch, Peutz-Jeghers) or chronic intestinal inflammation (Crohn’s disease).	It usually involves a short intestinal segment, but wide resections with nutritional consequences may be required.
Lymphoma	It depends on the type, different location. In our environment, it is more frequent in terminal ileum. It is considered primary intestinal lymphoma if there are no other adenopathy, except regional ones and the extension of the peripheral blood, liver and spleen are normal.	Similar to other neoplasms of the small intestine, although it has a lower tendency to occlude the intestinal lumen and more to perforate.	Classically there were 3 types, but now this classification is under review. Most frequent lymphoma B. There is lymphoid tissue lymphoma associated with mucosa (MALT, immunoproliferative disease of the small intestine (EIPID), Mediterranean lymphoma); T-cell lymphoma associated with celiac disease and other types of lymphomas, including a subtype considered benign, located in the duodenum.	Your relative risk increases in Crohn’s disease, celiac disease not following a gluten-free diet, HIV infection, or organ transplantation.	In some types, the condition is usually more extensive and can lead to greater nutritional consequences (clinically manifest intestinal malabsorption, sometimes associated with bacterial overgrowth).
GIST (gastrointestinal stromal tumors)	Jejunum and ileum. They are derived from the interstitial cells of Cajal.	Extraluminal growth, although they tend to ulcerate. They can be large in size. Abdominal pain, bleeding, perforation, or palpable abdominal mass.	It is characterized molecularly by mutations in the KIT proto-oncogene, with overexpression of c-kit (receptor tyrosine kinase detectable by immunohistochemistry). Before knowing these molecular characteristics they were confused with leiomyosarcomas.	Technically it is a soft tissue sarcoma, but totally different from any other variety. It should always be considered as potentially malignant, especially small bowel GISTs.	Not specific. Tyrosine kinase inhibitors have been a revolution in avoiding extensive intestinal resections of the past, with potentially dire nutritional consequences.
Tumor carcinoide	Terminal ileum, where they are the most frequent tumor. Other locations are possible	When they cause symptoms, they are the usual ones of small intestine tumors or the specific carcinoid syndrome.	They are usually small, sometimes multifocal, usually subepithelial and with a tendency to affect the serosa, with a marked desmoplastic reaction, visible on imaging techniques.	Those located in the appendix can debut as appendicitis.	They can produce carcinoid syndrome, sometimes after triggers. Vitamin deficiencies (pellagra and others) or other nutrients (see text and Table 8).

**Table 9:** Possible “specific” nutritional or metabolic alterations produced by digestive neuroendocrine tumors.

Tumor Type	Nutritional Or Metabolic Changes
All	Malnutrition
Insulinoma	Hypoglycemia, obesity
Glucagonoma	Hyperglycemia
Somatostatinoma	Hyperglycemia, protein deficits, Zn deficiency, fat-soluble vitamin deficiencies
VIPoma	Diarrhea, hypokalemia, hypomagnesemia, Zn deficiency
Gastrinoma	Diarrhea due to steatorrhea, hypomagnesemia, other electrolyte disturbances
Carcinoid tumor	Diarrhea, dehydration, niacin deficiency (also possible in fat-soluble vitamins), hypokalemia, hypomagnesemia, protein deficits

NOTE: the treatment of these tumors can produce associated secondary alterations, such as bile salt malabsorption, bacterial overgrowth, and, in the case of pancreatic tumors, pancreatic insufficiency.

## References

[1] Gee M.I., Grace M.G., Wensel R.H., Sherbaniuk R., Thomson A.B. (1985). Protein-energy malnutrition in gastroenterology outpatients: Increased risk in Crohn’s disease. J. Am. Diet. Assoc..

[2] Casanova M.J., Chaparro M., Molina B., Merino O., Batanero R., Dueñas-Sadornil C., Robledo P., Garcia-Albert A.M., Gómez-Sánchez M.B., Calvet X. (2017). Prevalence of malnutrition and nutritional characteristics of patients with inflammatory bowel disease. J. Crohn’s Colitis.

[3] Cabré E., Gassull M.A. (2001). Nutrition in inflammatory bowel disease: Impact on disease and therapy. Curr. Opin. Gastroenterol..

[4] Curtis L.J., Bernier P., Jeejeebhoy K., Allard J., Duerksen D., Gramlich L., Laporte M., Keller H.H. (2017). Costs of hospital malnutrition. Clin. Nutr..

[5] Cucino C., Sonnenberg A. (2001). Cause of death in patients with inflammatory bowel disease. Inflamm. Bowel Dis..

[6] Bozzetti F., Gianotti L., Braga M., di Carlo V., Mariani L. (2007). Postoperative complications in gastrointestinal cancer patients: The joint role of the nutritional status and the nutritional support. Clin. Nutr..

[7] Halmos E.P., Gibson P.R. (2015). Dietary management of IBD—Insights and advice. Nat. Rev. Gastroenterol. Hepatol..

[8] Narsale A.A., Carson J.A. (2014). Role of interleukin-6 in cachexia: Therapeutic implications. Curr. Opin. Supportive Palliat. Care.

[9] Patel H.J., Patel B.M. (2017). TNF-α and cancer cachexia: Molecular insights and clinical implications. Life Sci..

[10] Limdi J.K., Aggarwal D., McLaughlin J.T. (2015). Dietary practices and beliefs in patients with inflammatory bowel disease. Inflamm. Bowel Dis..

[11] Zallot C., Quilliot D., Chevaux J.B., Peyrin-Biroulet C., Guéant-Rodriguez R.M., Freling E., Collet-Fenetrier B., Williet N., Ziegler O., Bigard M.A. (2013). Dietary beliefs and behavior among inflammatory bowel disease patients. Inflamm. Bowel Dis..

[12] Grüner N., Mattner J. (2021). Bile acids and microbiota: Multifaceted and versatile regulators of the liver–gut axis. Int. J. Mol. Sci..

[13] Winter T.A., O’Keefe S.J.D.O., Callanan M., Marks T. (2004). Impaired gastric acid and pancreatic enzyme secretion in patients with Crohn’s disease may be a consequence of a poor nutritional state. Inflamm. Bowel Dis..

[14] Lee S.H. (2015). Intestinal permeability regulation by tight junction: Implication on inflammatory bowel diseases. Intest. Res..

[15] Al-Jaouni R., Hébuterne X., Pouget I., Rampal P. (2000). Energy metabolism and substrate oxidation in patients with Crohn’s disease. Nutrition.

[16] Mingrone G., Capristo E., Greco A.v., Benedetti G., de Gaetano A., Tataranni P.A., Gasbarrini G. (1999). Elevated diet-induced thermogenesis and lipid oxidation rate in Crohn disease. Eur. J. Gastroenterol. Hepatol..

[17] Casanova M.J., Ber Y., Montoro M., Bernal V. (2017). Impacto de la enfermedad inflamatoria intestinal sobre el estado nutricional. Consecuencias Nutricionales de las Enfermedades Digestivas. Clínicas Iberoamericanas de Gastroenterología y Hepatología.

[18] Perkal M.F., Seashore J.H. (1989). Nutrition and inflammatory bowel disease. Gastroenterol. Clin. North Am..

[19] Beeken W.L. (1973). Absorptive defects in young people with regional enteritis. Pediatrics.

[20] Sasson A.N., Ingram R.J.M., Raman M., Ananthakrishnan A.N. (2021). Nutrition in the management of inflammatory bowel diseases. Gastroenterol. Clin. North Am..

[21] Rossi R.E., Whyand T., Murray C.D., Hamilton M.I., Conte D., Caplin M.E. (2016). The role of dietary supplements in inflammatory bowel disease: A systematic review. Eur. J. Gastroenterol. Hepatol..

[22] Mouli V.P., Ananthakrishnan A.N. (2014). Review article: Vitamin D and inflammatory bowel diseases. Aliment. Pharmacol. Ther..

[23] Duerksen D.R., Fallows G., Bernstein C.N. (2006). Vitamin B12 malabsorption in patients with limited ileal resection. Nutrition.

[24] Reenan J. (2006). Clinical manifestations of vitamin B-12 deficiency. Virtual Mentor.

[25] Dignass A.U., Gasche C., Bettenworth D., Birgegård G., Danese S., Gisbert J.P., Gomollon F., Iqbal T., Katsanos K., Koutroubakis I. (2015). European consensus on the diagnosis and management of iron deficiency and anaemia in inflammatory bowel diseases. J. Crohn’s Colitis.

[26] Kumar A., Brookes M.J. (2020). Iron therapy in inflammatory bowel disease. Nutrients.

[27] Hartman C., Eliakim R., Shamir R. (2009). Nutritional status and nutritional therapy in inflammatory bowel diseases. World J. Gastroenterol..

[28] Seidman E., LeLeiko N., Ament M., Berman W., Caplan D., Evans J., Kocoshis S., Lake A., Motil K., Sutphen J. (1991). Nutritional issues in pediatric inflammatory bowel disease. J. Pediatr. Gastroenterol. Nutr..

[29] Dumitrescu G., Mihai C., Dranga M., Prelipcean C.C. (2013). Bone mineral density in patients with inflammatory bowel disease from North-Eastern Romania. Rev. Med. Chir. Soc. Med. Nat. Iasi..

[30] Laakso S., Valta H., Verkasalo M., Toiviainen-Salo S., Mäkitie O. (2014). Compromised peak bone mass in patients with inflammatory bowel disease—A prospective study. J. Pediatr..

[31] Kim H.J., Hong S.J., Jeon Y.W., Han J.P., Han S.H., Kang J.H., Tae J.W., Lim H.S., Kim H.K., Ko B.M. (2013). The early onset of disease may be a risk factor for decreased bone mineral density in patients with inflammatory bowel disease. Clin. Endosc..

[32] Abraham B.P., Prasad P., Malaty H.M. (2014). Vitamin D deficiency and corticosteroid use are risk factors for low bone mineral density in inflammatory bowel disease patients. Dig. Dis. Sci..

[33] Miznerova E., Hlavaty T., Koller T., Toth J., Holociova K., Huorka M., Killinger Z., Payer J. (2013). The prevalence and risk factors for osteoporosis in patients with inflammatory bowel disease. Bratisl. Lek. Listy.

[34] Bryant R.v., Trott M.J., Bartholomeusz F.D., Andrews J.M. (2013). Systematic review: Body composition in adults with inflammatory bowel disease. Aliment. Pharmacol. Ther..

[35] Jahnsen J., Falch J.A., Mowinckel P., Aadland E. (2003). Body composition in patients with inflammatory bowel disease: A population-based study. Am. J. Gastroenterol..

[36] Yao G.X., Wang X.R., Jiang Z.M., Zhang S.Y., Ni A.P. (2005). Role of perioperative parenteral nutrition in severely malnourished patients with Crohn’s disease. World J. Gastroenterol..

[37] Stokes M.A. (1992). Crohn’s disease and nutrition. Br. J. Surg..

[38] Valentini L., Schaper L., Buning C., Hengstermann S., Koernicke T., Tillinger W., Guglielmi F.W., Norman K., Buhner S., Ockenga J. (2008). Malnutrition and impaired muscle strength in patients with Crohn’s disease and ulcerative colitis in remission. Nutrition.

[39] Cederholm T., Bosaeus I., Barazzoni R., Bauer J., van Gossum A., Klek S., Muscaritoli M., Nyulasi I., Ockenga J., Schneider S.M. (2015). Diagnostic criteria for malnutrition—An ESPEN consensus statement. Clin. Nutr..

[40] Li S., Ney M., Eslamparast T., Vandermeer B., Ismond K.P., Kroeker K., Halloran B., Raman M., Tandon P. (2019). Systematic review of nutrition screening and assessment in inflammatory bowel disease. World J. Gastroenterol..

[41] Detsky A.S., Baker J.P., Mendelson R.A., Wolman S.L., Wesson D.E., Jeejeebhoy K.N., Detsky A.S., Baker J.P., Mendelson R.A., Wolman S.L. (1984). Evaluating the accuracy of nutritional assessment techniques applied to hospitalized patients: Methodology and comparisons. JPEN.

[42] Van Bokhorst-de van der Schueren M.A.E., Guaitoli P.R., Jansma E.P., de Vet H.C.W. (2014). Nutrition screening tools: Does one size fit all? A systematic review of screening tools for the hospital setting. Clin. Nutr..

[43] Sanderson I.R., Croft N.M. (2005). The anti-inflammatory effects of enteral nutrition. JPEN.

[44] Gassull M.À., Cabré E., Gassull M.À., Gomollón F., Hinojosa J., Obrador A. (2007). Nutrición y Enfermedad Inflamatoria Intestinal. Enfermedad inflamatoria intestinal.

[45] Shah N.D., Parian A.M., Mullin G.E., Limketkai B.N. (2015). Oral diets and nutrition support for inflammatory bowel disease: What is the evidence?. Nutr. Clin. Pract..

[46] Lochs H., Dejong C., Hammarqvist F., Hebuterne X., Leon-Sanz M., Schütz T., van Gemert W., van Gossum A., Valentini L., Lübke H. (2006). ESPEN guidelines on enteral nutrition: Gastroenterology. Clin. Nutr..

[47] Gomollón F., Dignass A., Annese V., Tilg H., van Assche G., Lindsay J.O., Peyrin-Biroulet L., Cullen G.J., Daperno M., Kucharzik T. (2017). 3rd European evidence-based consensus on the diagnosis and management of Crohn’s disease 2016: Part 1: Diagnosis and medical management. J. Crohns Colitis.

[48] Comeche J.M., Caballero P., Gutierrez-Hervas A., Garc S., Comino I., Altavilla C., Tuells J. (2019). Enteral nutrition in patients with inflammatory bowel disease. Systematic review, meta-analysis, and meta-regression. Nutrients.

[49] Van Gossum A., Cabre E., Hébuterne X., Jeppesen P., Krznaric Z., Messing B., Powell-Tuck J., Staun M., Nightingale J. (2009). ESPEN guidelines on parenteral nutrition: Gastroenterology. Clin. Nutr..

[50] Navas-López V.M., van Limbergen J., Martín-de-Carpi J. (2016). Nutrición enteral en el paciente pediátrico con enfermedad de Crohn. Enferm. Inflamatoria Intest. Dia.

[51] Dziechciarz P., Horvath A., Shamir R., Szajewska H. (2007). Meta-analysis: Enteral nutrition in active Crohn’s disease in children. Aliment. Pharmacol. Ther..

[52] Narula N., Dhillon A., Zhang D., Sherlock M.E., Tondeur M., Zachos M. (2018). Enteral nutritional therapy for induction of remission in Crohn’s disease. Cochrane Database Syst. Rev..

[53] Zachos M., Tondeur M., Griffiths A.M. (2007). Enteral nutritional therapy for induction of remission in Crohn’s disease. Cochrane Database Syst. Rev..

[54] Hu D., Ren J., Wang G., Li G., Liu S., Yan D., Gu G., Zhou B., Wu X., Chen J. (2014). Exclusive enteral nutritional therapy can relieve inflammatory bowel stricture in Crohn’s disease. J. Clin. Gastroenterol..

[55] Dignass A., van Assche G., Lindsay J.O., Lémann M., Söderholm J., Colombel J.F., Danese S., d’Hoore A., Gassull M., Gomollón F. (2010). The second European evidence-based consensus on the diagnosis and management of Crohn’s disease: Current management. J. Crohns Colitis.

[56] Uchino M., Ikeuchi H., Matsuoka H., Matsumoto T., Takesue Y., Tomita N. (2012). Clinical features and management of duodenal fistula in patients with Crohn’s disease. Hepato Gastroenterol..

[57] Mowat C., Cole A., Windsor A., Ahmad T., Arnott I., Driscoll R., Mitton S., Orchard T., Rutter M., Younge L. (2011). Guidelines for the management of inflammatory bowel disease in adults. Gut.

[58] Ravindran P., Ansari N., Young C.J., Solomon M.J. (2014). Definitive surgical closure of enterocutaneous fistula: Outcome and factors predictive of increased postoperative morbidity. Colorectal Dis..

[59] Triantafillidis J.K., Papalois A.E. (2014). The role of total parenteral nutrition in inflammatory bowel disease: Current aspects. Scand. J. Gastroenterol..

[60] Weimann A., Braga M., Carli F., Higashiguchi T., Hübner M., Klek S., Laviano A., Ljungqvist O., Lobo D.N., Martindale R. (2017). ESPEN guideline: Clinical nutrition in surgery. Clin. Nutr..

[61] Pironi L., Arends J., Bozzetti F., Cuerda C., Gillanders L., Jeppesen P.B., Joly F., Kelly D., Lal S., Staun M. (2016). ESPEN guidelines on chronic intestinal failure in adults. Clin. Nutr..

[62] Frolkis A.D., Dykeman J., Negrón M.E., Debruyn J., Jette N., Fiest K.M., Frolkis T., Barkema H.W., Rioux K.P., Panaccione R. (2013). Risk of surgery for inflammatory bowel diseases has decreased over time: A systematic review and meta-analysis of population-based studies. Gastroenterology.

[63] Alós R., Hinojosa J. (2008). Timing of surgery in Crohn’s disease: A key issue in the management. World J. Gastroenterol..

[64] Gillis C., Carli F. (2015). Promoting perioperative metabolic and nutritional care. Anesthesiology.

[65] Mainous M.R., Deitch E.A. (1994). Nutrition and infection. Surg. Clin. North Am..

[66] Santos J.I. (1994). Nutrition, infection, and immunocompetence. Infect. Dis. Clin. North Am..

[67] Stechmiller J.K. (2010). Understanding the role of nutrition and wound healing. Nutr. Clin. Pract..

[68] Brennan G.T., Ha I., Hogan C., Nguyen E., Jamal M.M., Bechtold M.L., Nguyen D.L. (2018). Does preoperative enteral or parenteral nutrition reduce postoperative complications in Crohn’s disease patients: A meta-analysis. Eur. J. Gastroenterol. Hepatol..

[69] Brady M., Kinn S., Stuart P. (2003). Preoperative fasting for adults to prevent perioperative complications. Cochrane Database Syst. Rev..

[70] Osland E., Yunus R.M., Khan S., Memon M.A. (2011). Early versus traditional postoperative feeding in patients undergoing resectional gastrointestinal surgery: A meta-analysis. JPEN.

[71] Ljungqvist O. (2016). Insulin resistance in surgery. Cir. Cir..

[72] Ljungqvist O. (2014). ERAS—Enhanced recovery after surgery: Moving evidence-based perioperative care to practice. JPEN.

[73] Dutheil F., Lac G., Lesourd B., Chapier R., Walther G., Vinet A., Sapin V., Verney J., Ouchchane L., Duclos M. (2013). Different modalities of exercise to reduce visceral fat mass and cardiovascular risk in metabolic syndrome: The RESOLVE randomized trial. Int. J. Cardiol..

[74] Bischoff S.C., Escher J., Hébuterne X., Kłęk S., Krznaric Z., Schneider S., Shamir R., Stardelova K., Wierdsma N., Wiskin A.E. (2020). ESPEN practical guideline: Clinical nutrition in inflammatory bowel disease. Clin. Nutr..

[75] Pironi L., Arends J., Baxter J., Bozzetti F., Peláez R.B., Cuerda C., Forbes A., Gabe S., Gillanders L., Holst M. (2015). ESPEN endorsed recommendations: Definition and classification of intestinal failure in adults. Clin. Nutr..

[76] Joly F., Baxter J., Staun M., Kelly D.G., Hwa Y.L., Corcos O., de Francesco A., Agostini F., Klek S., Santarpia L. (2018). Five-year survival and causes of death in patients on home parenteral nutrition for severe chronic and benign intestinal failure. Clin. Nutr..

[77] Massironi S., Cavalcoli F., Rausa E., Invernizzi P., Braga M., Vecchi M. (2020). Understanding short bowel syndrome: Current status and future perspectives. Dig. Liver Dis..

[78] Kelly D.G., Tappenden K.A., Winkler M.F. (2014). Short bowel syndrome: Highlights of patient management, quality of life, and survival. JPEN.

[79] Messing B., Pigot F., Rongier M., Morin M.C., Ndeïndoum U., Rambaud J.C. (1991). Intestinal absorption of free oral hyperalimentation in the very short bowel syndrome. Gastroenterology.

[80] Nightingale J.M.D., Kamm M.A., van der Sijp J.R.M., Ghatei M.A., Bloom S.R., Lennard-Jones J.E. (1996). Gastrointestinal hormones in short bowel syndrome. Peptide YY may be the “colonic brake” to gastric emptying. Gut.

[81] Crenn P., Coudray-Lucas C., Thuillier F., Cynober L., Messing B. (2000). Postabsorptive plasma citrulline concentration is a marker of absorptive enterocyte mass and intestinal failure in humans. Gastroenterology.

[82] Crenn P., Vahedi K., Lavergne-Slove A., Cynober L., Matuchansky C., Messing B. (2003). Plasma citrulline: A marker of enterocyte mass in villous atrophy-associated small bowel disease. Gastroenterology.

[83] Nightingale J., Woodward J.M. (2006). Guidelines for management of patients with a short bowel. Gut.

[84] Pironi L. (2016). Definitions of intestinal failure and the short bowel syndrome. Best Pract. Res. Clin. Gastroenterol..

[85] Kowlgi N.G., Chhabra L. (2015). D-lactic acidosis: An underrecognized complication of short bowel syndrome. Gastroenterol. Res. Pract..

[86] Bikle D.D. (2007). Vitamin D insufficiency/deficiency in gastrointestinal disorders. J. Bone Miner. Res..

[87] Khaodhiar L., Keane-Ellison M., Tawa N.E., Thibault A., Burke P.A., Bistrian B.R. (2002). Iron deficiency anemia in patients receiving home total parenteral nutrition. JPEN.

[88] Hwa Y.L., Rashtak S., Kelly D.G., Murray J.A. (2016). Iron deficiency in long-term parenteral nutrition therapy. JPEN.

[89] Nightingale J.M.D., Lennard-Jones J.E., Gertner D.J., Wood S.R., Bartram C.I. (1992). Colonic preservation reduces need for parenteral therapy, increases incidence of renal stones, but does not change high prevalence of gall stones in patients with a short bowel. Gut.

[90] Pironi L., Sasdelli A.S. (2019). Intestinal failure-associated liver disease. Clin. Liver Dis..

[91] Bond A., Huijbers A., Pironi L., Schneider S.M., Wanten G., Lal S. (2019). Review article: Diagnosis and management of intestinal failure-associated liver disease in adults. Aliment. Pharmacol. Ther..

[92] Amiot A., Messing B., Corcos O., Panis Y., Joly F. (2013). Determinants of home parenteral nutrition dependence and survival of 268 patients with non-malignant short bowel syndrome. Clin. Nutr..

[93] Bischoff S.C., Austin P., Boeykens K., Chourdakis M., Cuerda C., Jonkers-Schuitema C., Lichota M., Nyulasi I., Schneider S.M., Stanga Z. (2020). ESPEN guideline on home enteral nutrition. Clin. Nutr..

[94] Pironi L., Boeykens K., Bozzetti F., Joly F., Klek S., Lal S., Lichota M., Mühlebach S., van Gossum A., Wanten G. (2020). ESPEN guideline on home parenteral nutrition. Clin. Nutr..

[95] Buchman A.L., Fryer J., Wallin A., Ahn C.W., Polensky S., Zaremba K. (2006). Clonidine reduces diarrhea and sodium loss in patients with proximal jejunostomy: A controlled study. JPEN.

[96] Sato D., Morino K., Ohashi N., Ueda E., Ikeda K., Yamamoto H., Ugi S., Yamamoto H., Araki S., Maegawa H. (2013). Octreotide improves early dumping syndrome potentially through incretins: A case report. Endocr. J..

[97] Nightingale J.M., Walker E.R., Farthing M.J., Lennard-Jones J.E. (1991). Effect of omeprazole on intestinal output in the short bowel syndrome. Aliment. Pharmacol. Ther..

[98] Jeppesen P.B., Sanguinetti E.L., Buchman A., Howard L., Scolapio J.S., Ziegler T.R., Gregory J., Tappenden K.A., Holst J., Mortensen P.B. (2005). Teduglutide (ALX-0600), a dipeptidyl peptidase IV resistant qlucagon-like peptide 2 analogue, improves intestinal function in short bowel syndrome patients. Gut.

[99] Jeppesen P.B., Pertkiewicz M., Messing B., Iyer K., Seidner D.L., O’Keefe S.J.D., Forbes A., Heinze H., Joelsson B. (2012). Teduglutide reduces need for parenteral support among patients with short bowel syndrome with intestinal failure. Gastroenterology.

[100] Yannam G.R., Sudan D.L., Grant W., Botha J., Langnas A., Thompson J.S. (2010). intestinal lengthening in adult patients with short bowel syndrome. J. Gastrointest. Surg..

[101] Dutta S. (2007). The STEP procedure: Defining its role in the management of pediatric short bowel syndrome. J. Pediatr. Gastroenterol. Nutr..

[102] Frongia G., Kessler M., Weih S., Nickkholgh A., Mehrabi A., Holland-Cunz S. (2013). Comparison of LILT and STEP procedures in children with short bowel syndrome—A systematic review of the literature. J. Pediatr. Surg..

[103] Knoyle P.-M. (2021). Treating SBS with organoids. Nat. Rev. Gastroenterol. Hepatol..

[104] Sugimoto S., Kobayashi E., Fujii M., Ohta Y., Arai K., Matano M., Ishikawa K., Miyamoto K., Toshimitsu K., Takahashi S. (2021). An organoid-based organ-repurposing approach to treat short bowel syndrome. Nature.

[105] Cancer Stat Facts: Colorectal Cancer. https://seer.cancer.gov/statfacts/html/colorect.html.

[106] Zhou E., Rifkin S. (2021). Colorectal cancer and diet: Risk versus prevention, is diet an intervention?. Gastroenterol. Clin. North Am..

[107] Keum N.N., Giovannucci E. (2019). Global burden of colorectal cancer: Emerging trends, risk factors and prevention strategies. Nat. Rev. Gastroenterol. Hepatol..

[108] Veettil S.K., Wong T.Y., Loo Y.S., Playdon M.C., Lai N.M., Giovannucci E.L., Chaiyakunapruk N. (2021). Role of diet in colorectal cancer incidence: Umbrella review of meta-analyses of prospective observational studies. JAMA Netw. Open.

[109] Stoffel E.M., Murphy C.C. (2020). Epidemiology and mechanisms of the increasing incidence of colon and rectal cancers in young adults. Gastroenterology.

[110] Paski S.C., Semrad C.E. (2009). Small bowel tumors. Gastrointest. Endosc. Clin. North Am..

[111] Barsouk A., Rawla P., Barsouk A., Thandra K.C. (2019). Epidemiology of cancers of the small intestine: Trends, risk factors, and prevention. Med. Sci..

[112] Howe J.R., Cardona K., Fraker D.L., Kebebew E., Untch B.R., Wang Y.Z., Law C.H., Liu E.H., Kim M.K., Menda Y. (2017). The surgical management of small bowel neuroendocrine tumors. Pancreas.

[113] Laing E., Kiss N., Michael M., Krishnasamy M., Laing M.E. (2019). At the cutting edge nutritional complications and the management of patients with gastroenteropancreatic neuroendocrine tumors. Neuroendocrinology.

[114] Clement D.S.V.M., Tesselaar M.E.T., van Leerdam M.E., Srirajaskanthan R., Ramage J.K. (2019). Nutritional and vitamin status in patients with neuroendocrine neoplasms. World J. Gastroenterol..

[115] Williams M.L., Torres-Duarte A., Brant L.J., Bhargava P., Marshall J., Wainer I.W. (2004). The relationship between a urinary cachectic factor and weight loss in advanced cancer patients. Cancer Investig..

[116] Van Cutsem E., Arends J. (2005). The causes and consequences of cancer-associated malnutrition. Eur. J. Oncol. Nurs..

[117] Russell S.T., Tisdale M.J. (2002). Effect of a tumour-derived lipid-mobilising factor on glucose and lipid metabolism in vivo. Br. J. Cancer.

[118] Kasprzak A. (2021). The role of tumor microenvironment cells in colorectal cancer (CRC) cachexia. Int. J. Mol. Sci..

[119] Dolan R.D., Laird B.J.A., Klepstad P., Kaasa S., Horgan P.G., Paulsen Ø., McMillan D.C. (2019). An exploratory study examining the relationship between performance status and systemic inflammation frameworks and cytokine profiles in patients with advanced cancer. Medicine.

[120] Bowers L., Boyle D.A. (2003). Depression in patients with advanced cancer. Clin. J. Oncol. Nurs..

[121] Nelson K.A. (2000). The cancer anorexia-cachexia syndrome. Semin. Oncol..

[122] Wallengren O., Lundholm K., Bosaeus I. (2013). Diagnostic criteria of cancer cachexia: Relation to quality of life, exercise capacity and survival in unselected palliative care patients. Support. Care Cancer.

[123] Barret M., Malka D., Aparicio T., Dalban C., Locher C., Sabate J.-M., Louafi S., Mansourbakht T., Bonnetain F., Attar A. (2011). Nutritional status affects treatment tolerability and survival in metastatic colorectal cancer patients: Results of an AGEO prospective multicenter study. Oncology.

[124] Gallois C., Artru P., Lièvre A., Auclin E., Lecomte T., Locher C., Marthey L., Zaimi Y., Faroux R., Pernot S. (2019). Evaluation of two nutritional scores’ association with systemic treatment toxicity and survival in metastatic colorectal cancer: An AGEO prospective multicentre study. Eur. J. Cancer.

[125] Karabulut S., Dogan I., Usul Afsar C., Karabulut M., Ak N., Duran A., Tastekin D. (2021). Does nutritional status affect treatment tolerability, chemotherapy response and survival in metastatic gastric cancer patients? Results of a prospective multicenter study in Turkey. J. Oncol. Pharm. Pract..

[126] Nakajima N. (2021). Differential diagnosis of cachexia and refractory cachexia and the impact of appropriate nutritional intervention for cachexia on survival in terminal cancer patients. Nutrients.

